# An International Medical Congress

**Published:** 1891-06

**Authors:** Charles F. Deems, R. S. Cheves

**Affiliations:** President of the Park Association; Secretary, West New Brighton, N. Y.


### Correspondence.

### AN INTERNATIONAL MEDICAL CONGRESS.

The managers of the National Prohibition Park, of Staten Island, invite representative medical men from all localities in the United States and the Dominion of Canada to meet in conference on the 15th and 16th of July next, in the great Auditorium Building of the Park. The chief object of the meeting is to be the comparison of views on the relationship of physiology and alcohol. Among the questions to be discussed will be the following:

What are the Hereditary Effects of Drunkenness?

Are there any Hereditary Effects that Follow Moderate Drinking?

To What Diseases are Inebriates More Especially Exposed?

Is Alcohol a Poison?

Is Alcohol in Any Sense a Food?

What are the Proper Uses of Alcohol as a Medicine?

Is there any Danger of Producing the Drink Habit from the Prescribing of Alcoholic Medicines?

How Large a Percentage of Deaths May be Atributed, Directly or Indirectly, to the Use of Strong Drink?

Should Alcoholic Liquors Ever be Used Except under the Direction of a Medical Adviser?

Dr. Nathan S. Davis, of Chicago, will preside at the Congress, and will make the opening address. It will be remembered that Dr. Davis presided at the recent International Medical Congress, held in Washington. It is particularly requested that from each town throughout the country, and also from each ward of every city a delegation of three physicians be appointed to attend. Clergymen, W. C. T. U., and other temperance organizations are urged to consult with the physicians in their vicinity and see that their neighborhoods are represented at the Congress.

At this Conference all views will be given an impartial hearing. No restraint will be placed upon the discussion save that of the time limit. Many well-known medical men have already signified their willingness to participate in such a Conference. No harm, but much good, may come from this conference of views by leading physicians. The general subject is a most urgent one. An eminent New York physician, the late Dr. Willard Parker, in his preface to the Lectures of Dr. Richardson, of London, said:

"Alcohol has no place in the healthy system, but is an 'irritant poison', producing a diseased condition of body and mind. Statistics show that ten per cent. of the annual number of deaths in this country are due to alcohol; that fully thirty-five per cent. of our insane are so either directly or indirectly from its use; and that from seventy-five to ninety per cent. of the inmates of our penal and pauper institutions owe their condition to its influence. Besides this, we find that forty-five per cent. of the inmates of our asylums for idiots are the offspring of parents addicted to drink.

"Destroying, as it does, the will, the judgment and the moral sense, may we not with propriety consider it a cause of that low state of public and private integrity which permits, even in our very midst, the formation of those shameful combinations to defraud and steal, commonly known as 'rings?' . . . . If the habitual use of distilled liquors increases as rapidly within the opening century as it has during the one just ending, how sad the outlook! I can discern nothing in the future but a wreck of national honor, and the sinking to a lower standard of civilizction and morality, unless public sentiment in this regard be changed."

Some time since, Mr. Gladstone, in a speech in the House of Common, declared that the drink evil in England has wrought more mischief than had the three great historic sources, war, famine and pestilence combined. Certainly, a question which so involves the health and general well being of society as does this question of the use of alcoholic liquor should interest no class of our citizens more deeply than physicians.

The Congress will assemble at 10 a. m., July 15th and 16th, and continue its session for two days. It is expected that arrangements will be made for reduced railroad fares.

CHARLES F. DEEMS,
President of the Park Association.

R. S. CHEVES, Secretary, West New Brighton, N. Y.

